# A West Antarctic grounding-zone environment shaped by episodic water flow

**DOI:** 10.1038/s41561-025-01687-3

**Published:** 2025-05-12

**Authors:** Huw J. Horgan, Craig Stewart, Craig Stevens, Gavin Dunbar, Linda Balfoort, Britney E. Schmidt, Peter Washam, Mauro A. Werder, Darcy Mandeno, James Marschalek, Christina Hulbe, Nicholas Holschuh, Richard Levy, Benjamin Hurwitz, Stefan Jendersie, Katelyn Johnson, Justin Lawrence, Regine Morgenstern, Andrew D. Mullen, Enrica Quartini, Wilson Sauthoff, Matthew Siegfried, Holly Still, Sam Thorpe-Loversuch, Tina van de Flierdt, Ryan Venturelli, Arran Whiteford

**Affiliations:** 1 Laboratory of Hydraulics, Hydrology and Glaciology (VAW), ETH Zurich, Zurich, Switzerland; 2 Swiss Federal Institute for Forest, Snow and Landscape Research (WSL), Sion, Switzerland; 3 Antarctic Research Centre, Victoria University of Wellington, Wellington, New Zealand; 4 Ocean Observations, National Institute for Water and Atmospheric Research, Wellington, New Zealand; 5 Department of Physics, University of Auckland, Auckland, New Zealand; 6 Department of Astronomy, Cornell University, Ithaca, NY USA; 7 Department of Earth and Atmospheric Sciences, Cornell University, Ithaca, NY USA; 8 School of Earth and Atmospheric Sciences, Georgia Institute of Technology, Atlanta, GA USA; 9 Department of Earth Science and Engineering, Imperial College London, London, UK; 10 School of Surveying, University of Otago, Dunedin, New Zealand; 11 Department of Geology, Amherst College, Amherst, MA USA; 12 GNS Science, Lower Hutt, New Zealand; 13 Honeybee Robotics, Altadena, CA USA; 14 Department of Geophysics, Colorado School of Mines, Golden, CO USA; 15 Department of Geology and Geological Engineering, Colorado School of Mines, Golden, CO USA

**Keywords:** Cryospheric science, Physical oceanography, Hydrology

## Abstract

Beneath Antarctica’s ice sheets, a little-observed network of liquid water connects vast landscapes and contributes to the motion of the overriding ice. When this subglacial water reaches the ocean cavity beneath ice shelves, it mixes with seawater, amplifying melt and in places forming deep channels in the base of the ice. Here we present observations from a hot-water-drilled borehole documenting subglacial water entering the ocean cavity at the grounding zone of Kamb Ice Stream and the Ross Ice Shelf. Our observations show that melt has removed approximately a third of the ice thickness, yet measurements reveal low rates of subglacial discharge in a turbid plume. Sediment cored from the channel floor shows larger discharge events occur and episodically deposit material from distinct geological domains. We quantify subglacial discharge and link our observations to the catchment upstream. We conclude that discrete discharge events are likely to dominate channel melt and sediment transport and result in the extensive ice-shelf features downstream of Kamb Ice Stream.

## Abstract (web summary)

Observations from a borehole in the Kamb Ice Stream suggest that discrete subglacial discharge events dominate channel melt and sediment transport.

## Main

Antarctica’s ice sheets are underlain by liquid water made possible by geothermal and frictional heat and the thick insulation provided by the ice itself^1^. This subglacial water facilitates the rapid sliding of the overriding ice^2^, transports sediment^3^, archives ice-sheet history^4^ and plays host to unique biological communities^5^. Subglacial water flows down hydraulic gradients determined mostly by the pressure exerted by the overlying ice^6^. In places, water pools in subglacial lakes, some of which fill and drain^7^ at times in a coordinated manner^8^. This variability is important as changing water discharge or its routing can change the movement of the overlying ice streams and glaciers at timescales of days to months^2^ to decades to centuries^9^. Eventually, subglacial water can reach the ice-sheet margin, emerging from beneath the grounded ice into the open ocean or a sub-ice-shelf cavity where it stimulates biological productivity^10^ and amplifies ice-shelf melt^11^.

When subglacial discharge reaches the ice-sheet–ice-shelf transition (the grounding zone) its conservative temperature (*Θ*) is close to its in situ freezing point (*Θ*_fp_ = −0.48 °C for freshwater at 650 m depth). Subglacial discharge therefore has little thermal driving (*Θ* − *Θ*_fp_ ≈ 0 °C) capable of generating ice melt on its own, but it is buoyant compared with the saline water it emerges into^12^. High-salinity shelf water (HSSW), which is generated by sea-ice formation at the ice-shelf front and circulates within the ice-shelf cavity, is by contrast dense and has a relatively high thermal driving (*Θ* – *Θ*_fp_ ≈ 0.5 °C at 650 m depth). The buoyancy of subglacial discharge and its ability to entrain HSSW means it can have an outsized effect on ice melt. By mixing with HSSW in the ice-shelf cavity and ascending the ice-shelf base, it can amplify ice melt^11,13^, in places forming deep channels in the base of ice shelves^14–16^. Channelized ice-shelf melt was first documented in Greenland^17^ and in the Antarctic can result in sub-ice-shelf channels, visible on the surface and often extending hundreds of kilometres^14,15^. This concentrated melt is thought to have both positive and negative effects on the ice shelves that buttress the flow of inland ice. Subglacial discharge is likely to disrupt the temperature-based stratification observed in the grounding-zone cavity, but this effect would be limited if discharge were confined to channels^18^. Modelled subglacial discharge correlates with observed ice-shelf mass loss^16^, which in turn reduces buttressing. Ice dynamic modelling including subglacial discharge shows that reduced buttressing and associated grounding-zone retreat can cause increased loss of grounded ice^19^. However, narrow channels have also been shown to reduce overall ice-shelf melt rates by concentrating melt^20^. Deeply incised channels can also promote fracture due to vertical flexing of the ice shelf^21,22^, and while ice flow may counter the effects of melt in thick ice, melt may destabilize thin ice shelves^23^.

Despite its importance, no direct observations of subglacial discharge have previously been made in the interior of Antarctica’s large cold-cavity ice shelves where HSSW contributes to melt^12^. Observations from beneath Thwaites eastern ice shelf in the Amundsen Sea Sector of West Antarctica recorded the sudden onset of subglacial discharge in mooring records^24^ while remotely operated vehicle observations documented subglacial discharge proximal to the mooring^25^. To our knowledge, the only other Antarctic observations of subglacial discharge come from East Antarctica, where subaerial discharge was observed at the ice-sheet margin^26^, and from Taylor Glacier, where a suspected remnant marine body discharges subaerially at Blood Falls^27^. Also noteworthy is the absence of subglacial discharge where it might be expected, such as close to grounding zones^28,29^ and at the downstream end of subglacial drainage paths^18^.

**Fig. 1:**
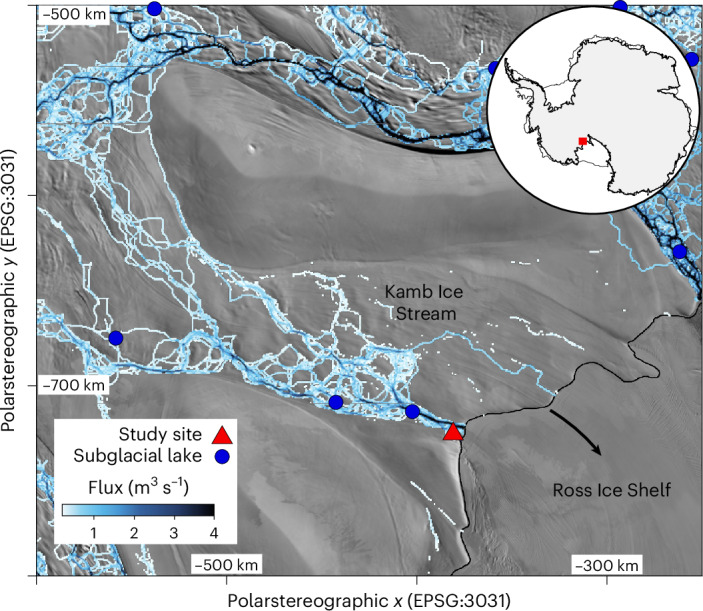
Study site. Subglacial water concentrates beneath KIS before entering the sub-ice-shelf cavity at the study site. Modelled subglacial water routing and flux (plotted in blue on the ice surface). Ice-sheet–ice-shelf transition (the grounding zone) shown by black line^35^ with black arrow showing general ice-flow direction. Background imagery from ref. ^50^. Source data

In this Article, we report observations of subglacial discharge crossing the grounding zone of Kamb Ice Stream (KIS) in West Antarctica (Fig. 1). KIS is one of the five main ice streams that feed ice into the Ross Ice Shelf from West Antarctica and is of particular interest as its rapid ice flow ceased approximately 190 years ago^30^. Currently, mass gain upstream on KIS offsets approximately a quarter of the mass loss occurring elsewhere in West Antarctica^31^, making the variability of KIS’s ice flow an important component of the West Antarctic Ice Sheet’s mass balance. Such stagnation and reactivation of ice streams has occurred in the past^32^ and has been attributed to changing basal thermal regime^9^, or the quantity and routing of water at the base of the ice^33^.

To observe subglacial discharge, we drilled a hot-water borehole through the ice in the austral summer of 2021–2022 at 82.4704° S, 152.2919° W (red triangle, Fig. 1). Our borehole accessed a prominent channel incised into the base of the ice approximately 750 m downstream from where subglacial water first interacts with seawater from the Ross Ice Shelf cavity^34^ and approximately 5 km upstream of the grounding line^35^. Melting in this grounding-zone channel has resulted in an upstream migration of the channel’s surface expression of 1.5 km since 1985^34,36^. The feature is geographically continuous with a substantial feature on the ice-shelf surface downstream^14,37^ (Fig. 1). Borehole access allowed us to image the channel interior and estimate the discharge of subglacial water. We combine the oceanographic observations with a sedimentary record collected from the floor of the channel and new estimates of subglacial routing and downstream ice thickness. Together our findings provide a comprehensive view of subglacial water discharge from beneath KIS and evidence for larger discharge events in recent decades.

## Direct observations of subglacial discharge

**Fig. 2:**
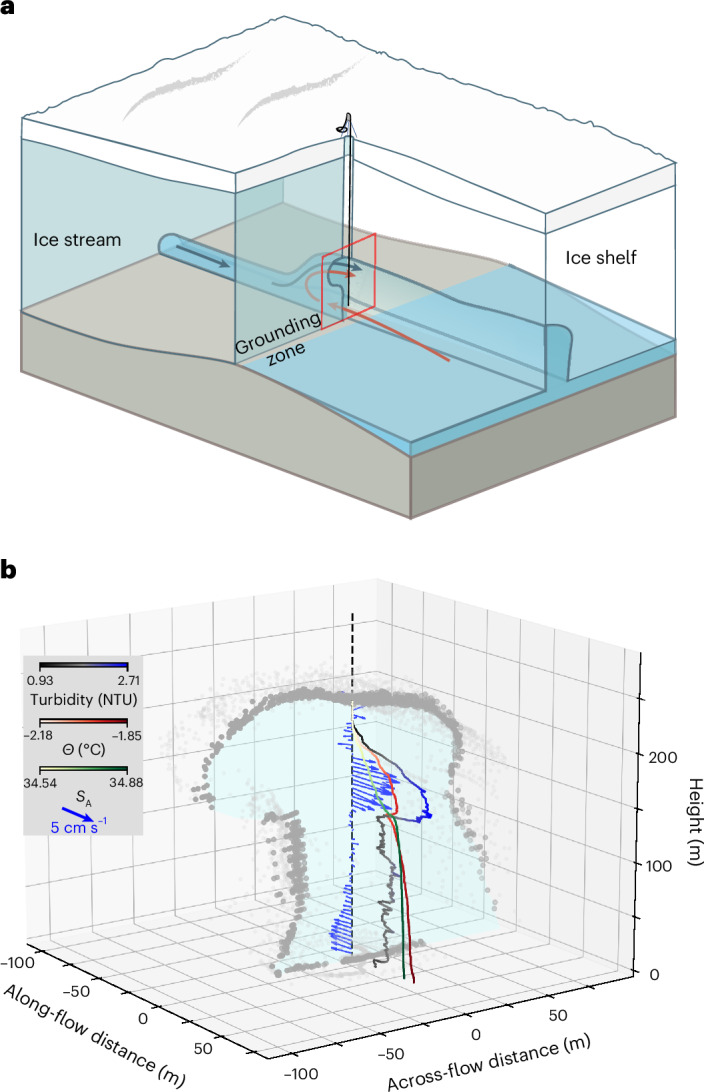
Borehole access. Hot-water drilling through approximately 500 m of ice accessed a water-filled channel approximately 250 m high. **a**, The borehole location downstream of where subglacial water (blue arrow) first interacts with ocean-cavity water (red arrow). The red parallelogram shows the location and orientation of **b**. **b**, Grounding-zone channel cross section (grey dots) imaged using a profiling acoustic sensor. Oceanographic observations, including velocity (blue vectors), turbidity, temperature and salinity, were profiled throughout the water column. Turbidity (grey–blue colour scale), temperature (red–white colour scale) and salinity (white–green colour scale) are normalized and plotted in the downstream (positive *x* axis) direction for illustrative purposes. Coordinates are metres in a local coordinate system (*x*, along channel; *y*, across channel; *z*, height above channel floor) with a vertical compression of approximately 2/1. Velocity vectors are scaled and plotted in the same coordinate system. The true bearing of the positive *x* axis is approximately 260°. Observations show estuarine-like flow whereby warmer saltier water flows upstream along the lower channel, and fresher cooler water travels down channel in a turbid plume in the lower half of the circular portion of the upper channel. NTU, nephelometric turbidity units. Source data

**Fig. 3:**
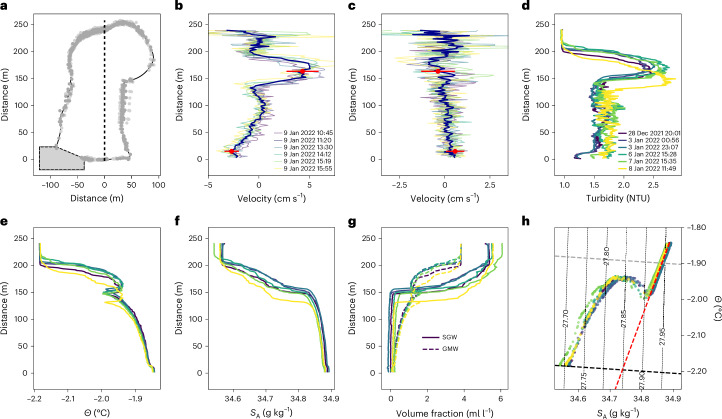
Water-column properties. A plume of turbid water flows downstream in the upper portion of the grounding-zone channel. **a**, Channel cross section looking downstream determined using borehole altimeter profiling. Grey box denotes region of uncertainty due to lack of altimeter returns. **b**,**c**, Longitudinal (**b**) and transverse (**c**) mean current (dark blue) and individual cast current profiles overlain with 14-day fixed mooring velocities (mean and standard deviation (variability) shown in red). Currents are positive downstream and to the right when looking downstream. Legend in **b** shows timing of cross-section and current observations. **d**, Turbidity for each cast session. Legend shows timing of turbidity, temperature and salinity observations. **e**, Conservative temperature (*Θ*) of each cast session. **f**, Absolute salinity (*S*_A_) of each cast session. **g**, Volume fraction of subglacial discharge water (SGW, solid lines) and glacial meltwater (GMW, dashed lines). **h**, Temperature–salinity values coloured by the same colour scale as **d**. Dashed red line in **h** denotes Gade line^38^. Heavy dashed black line denotes freezing point at the base of the ice (channel apex), while heavy dashed grey line denotes freezing point at sea surface. Dotted black lines denote contours of potential density anomaly (*σ*_0_). Source data

**Extended Data Fig. 1:**
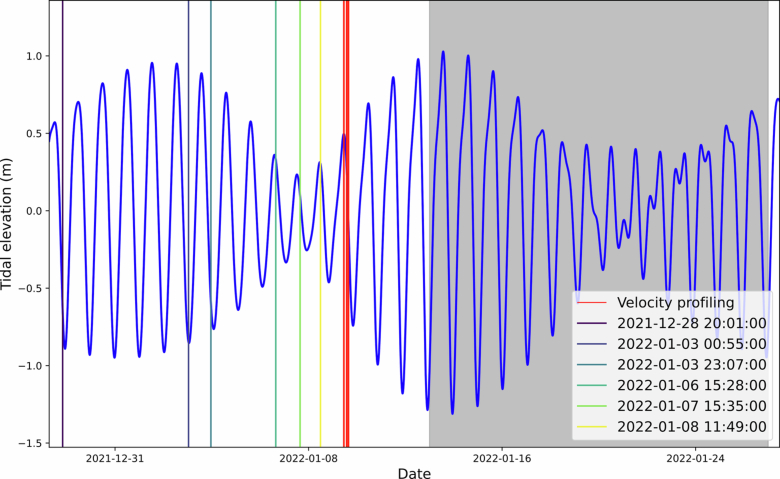
Experiment timing and tidal elevation. Experiment timing overlain on tidal elevation from CATS2008^2^ (blue line). Channel imaging and water column velocity timing is shown by red vertical lines. Oceanographic profiling for temperature, salinity, and turbidity timing is shown in the colours and detailed in the legend. Colours of oceanographic profiling match those in manuscript Fig. 2. Grey shaded area represents time period over which mooring current observations were averaged for comparison with profiling observations (Fig. 2). Source data

Beneath approximately 500 m of ice, we used an acoustic sensor to image a 252-m high water-filled grounding-zone channel (Fig. 2a). The channel had an oblate upper section approximately 107 m high overlying a narrower slot with near-vertical side extending 145 m down to the channel floor (Figs. 2b and 3a). Current velocities were measured during six vertical profiles over a 7-hour period, and six profiles of turbidity, temperature and salinity were measured over a 12-day period (Extended Data Fig. 1). These profiles revealed a layer of turbid water between 127 m and 218 m height above the channel floor (Figs. 2b and 3d). It flowed downstream and exhibited high vertical gradients in conservative temperature (*Θ*) and absolute salinity (*S*_A_) (hereafter, we refer to *Θ* and *S*_A_ as temperature and salinity). The highest rates of downstream flow reached 5 cm s^−1^ and coincided with the highest observed turbidity (Fig. 3b,d). Transverse flow velocities in the turbid layer were an order of magnitude lower than the longitudinal velocities (Fig. 3c). A relatively cold (*Θ* < –2.18 °C) and fresh (*S*_A_ < 34.57 g kg^–1^) low-turbidity layer was observed between the top of the channel and the high-turbidity outflow (Fig. 3d–f). This clear and well-mixed layer flowed longitudinally at low velocity both up- and downstream. Beneath the turbid outflow, within the vertical-walled section of the channel, low rates of downstream flow were observed, switching to an upstream flow of relatively warm and saline water in the lowermost 75 m of the channel. Peak upstream velocities reached almost 3 cm s^−1^ close to the channel floor where peak *Θ* and *S*_A_ were observed. Measured velocities in the turbid layer and lower channel were consistent with average velocity measured at fixed depths over a spring-neap tidal cycle (Fig. 3b,c).

**Extended Data Fig. 2:**
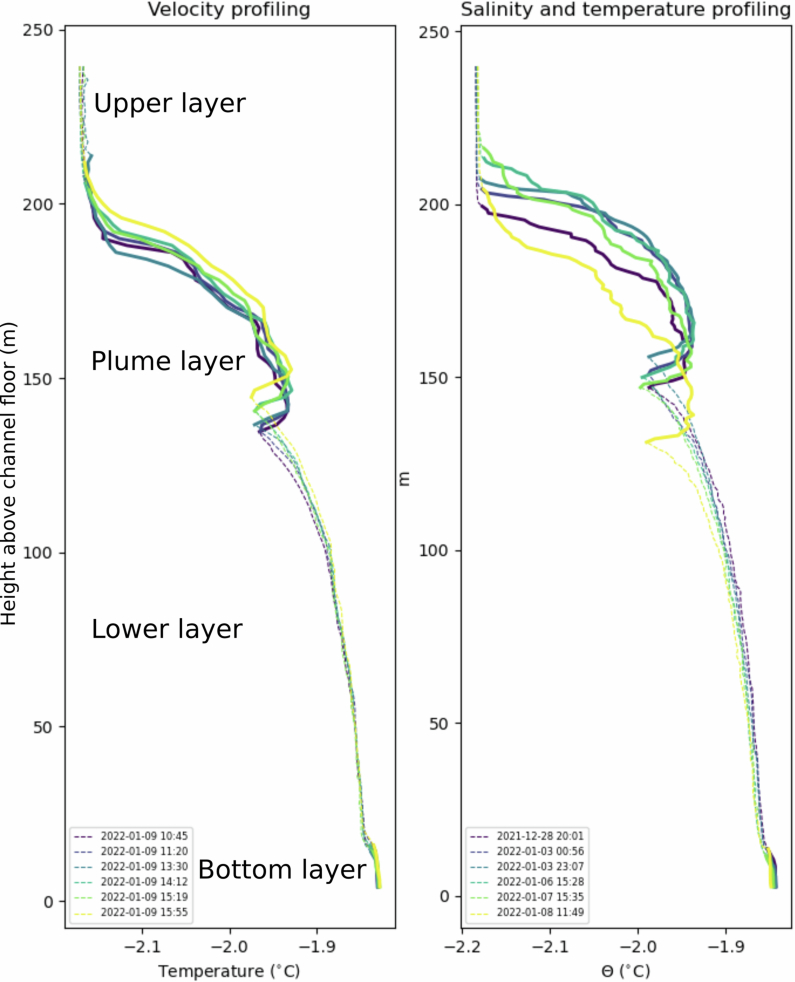
Water column temperature structure. Water column temperature structure during velocity profiling (left) and temperature and salinity profiling (right). Temperature based layer definitions from Supplementary Table 2 are shown using alternating dashed (upper and lower layers) and solid (plume and bottom layers) lines. Velocity profiling temperatures are shown as in-situ temperatures due to the absence of coincident salinity observations. Source data

We determined the sources of water in the channel using their distinct temperature and salinity values and a three-member mixing model^13^ (Extended Data Table 1, Supplementary Tables 1–5 and Extended Data Fig. 2) whereby oceanic source water is assumed to be well mixed with glacial meltwater (derived from ice melt within the grounding-zone channel) and subglacial discharge water (sourced from the subglacial network upstream) (Fig. 3g). In the case of freezing or the generation of glacial meltwater in well-mixed conditions, temperature and salinity evolve along a straight line in *Θ*–*S*_A_ space (a Gade line^38^). Deviation from the Gade-line gradient of 2.4 °C (g kg^–1^)^−1^ towards warmer, fresher conditions indicates subglacial discharge (Fig. 3h). Combining the channel cross section and longitudinal flow velocities and assuming uniform cross-channel properties allowed us to estimate subglacial discharge water flux through the channel of 0.9 ± 0.4 m^3^ s^−1^ (mean and standard deviation of observations). Glacial meltwater flux through the channel was similarly estimated as 0.3 ± 0.2 m^3^ s^−1^.

## Sediment record of episodic subglacial discharge

**Fig. 4:**
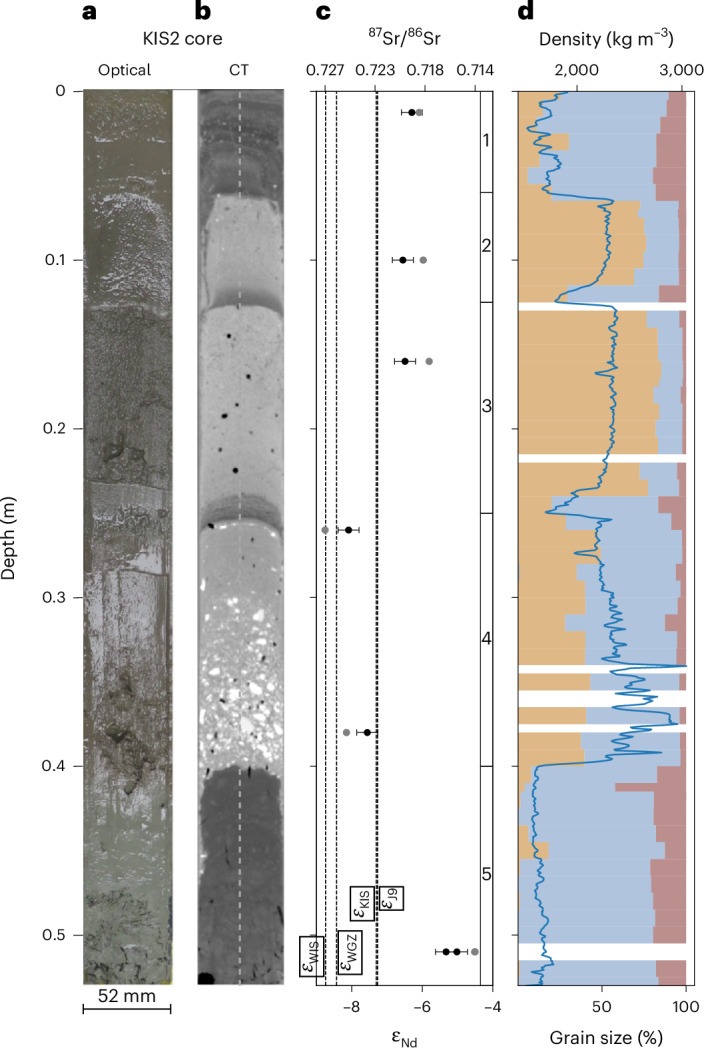
Sediment record. Sediment core recovered from the base of the channel shows sediment deposition that is dominated by distinct events. **a**, Visible image of split core. **b**, Single CT slice with dashed line showing location of density profile shown in **d**. **c**, Provenance indicators (neodymium isotope ratio *ε*_Nd_ in black, and ^87^Sr/^86^Sr in grey) with dashed lines denoting *ε*_Nd_ values from Whillans Grounding Zone (*ε*_WGZ_), Whillans Ice Stream (*ε*_WIS_), upstream Kamb Ice Stream (*ε*_KIS_) and J9 borehole (*ε*_J9_) (Supplementary Table 6). Note, *ε*_Nd_ and ^87^Sr/^86^Sr are estimated from single sample measurements. *ε*_Nd_ error bars are two standard deviations calculated from the external reproducibility of the JNdi standard, and ^87^Sr/^86^Sr error bars, estimated from the internal measurement error, are smaller than the symbol size. **d**, Sand (light brown), silt (light blue) and clay (dark brown) grain-size percentages overlain with density profile (blue line). Source data

**Extended Data Fig. 3:**
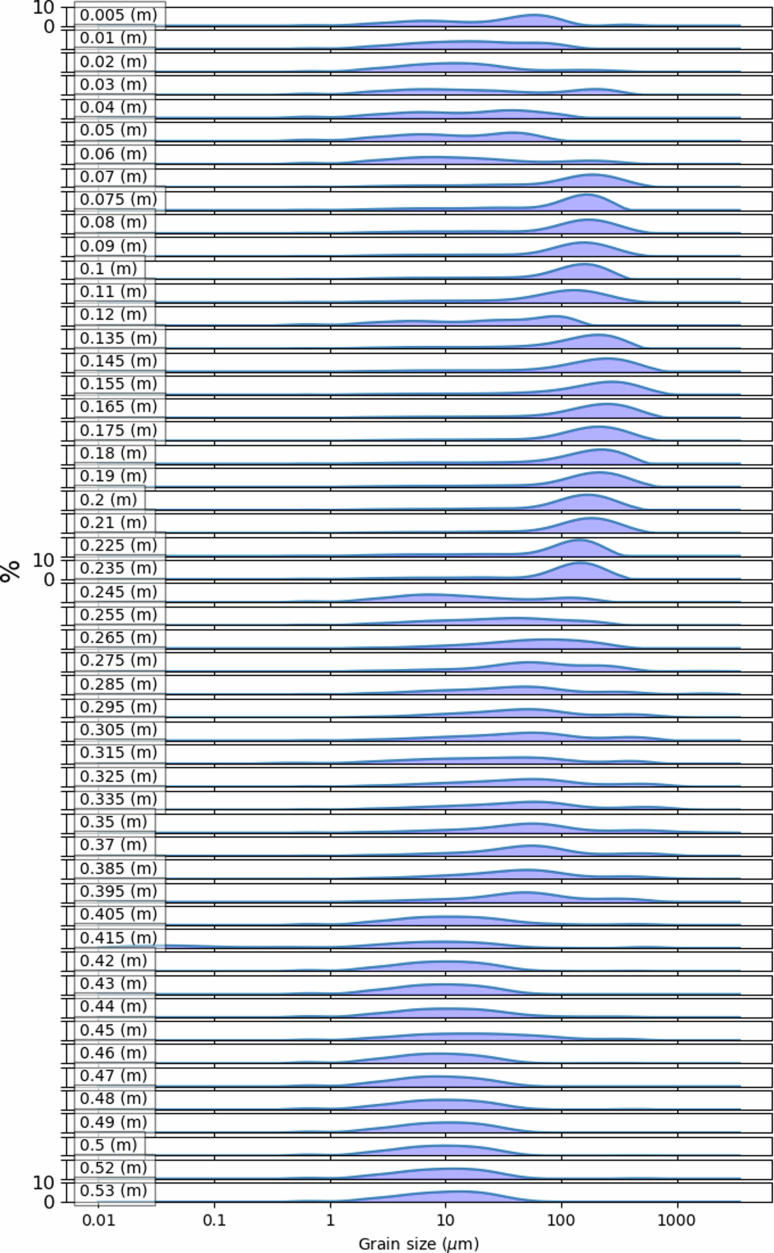
Grain size distribution. Grain size distributions for the < 1400 micro-m fraction at all depths measured in the KIS2 core. All y-axes limits are from 0 to 10 %. Annotations show sample depths which are not all equally spaced. Source data

Sedimentation in the channel has preserved a proxy record of subglacial discharge. This record is unlikely to provide a continuous record of drainage through the channel, with the possibility of erosional events, and variable rates of deposition. We recovered a 0.53-m-long sediment core consisting of five distinct units (Fig. 4 and Extended Data Fig. 3). The uppermost unit (0–6 cm) exhibits cm-scale sub-units characterized by light and dark couplets in computed tomography (CT) scans (Fig. 4b) and cycles in grain-size distribution and density (Fig. 4d). The couplets lack sharp transitions, implying continuous deposition. The sand content in this uppermost unit is low, with only a few coarse particles present, indicating deposition did not result from the melt-out of basal debris^39^. We also note the similarity between this unit and the rhythmite deposits recovered upstream on the adjacent Whillans Ice Stream^3^. We interpret this upper unit as resulting from subglacial discharge similar to that recorded by our oceanographic profiling and suggest the couplets result from flow variability. At times of higher flow, coarser material is transported and settles out and fines remain suspended, resulting in the darker deposits with lower bulk density. During lower flow periods, the finer material settles, resulting in the lighter-coloured deposits with higher bulk density.

Below 6 cm, two similar yet distinct units (units 2 and 3) are interpreted as deposits resulting from discrete discharge events. These deposits are bounded by abrupt contacts in CT scans (Fig. 4b), density and grain-size distribution (Fig. 4d). The contacts are depositional and show no evidence of erosion. Units 2 and 3 exhibit grain-size distributions that are indicative of higher flow speeds than the top 6 cm of the core with greater sand fractions. These units also exhibit inversely graded density profiles, commonly found in flow deposits with high sediment concentrations^40^.

**Fig. 5:**
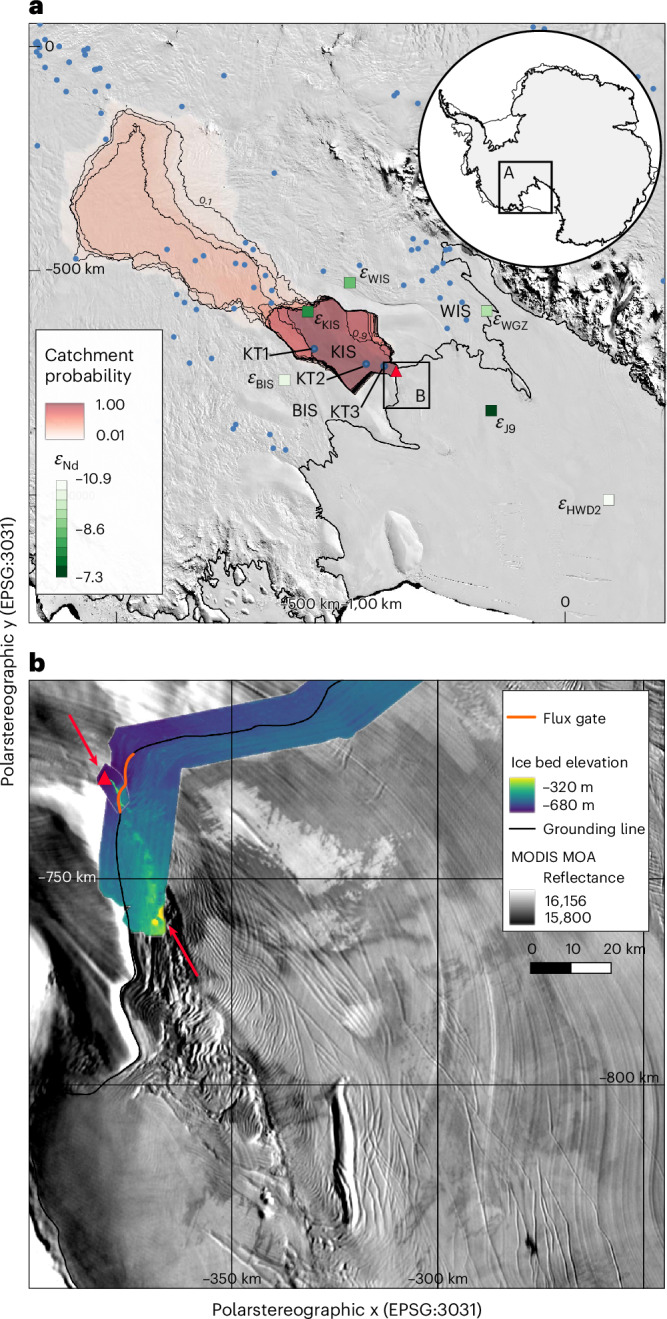
Upstream catchment and downstream impacts. **a**, Catchment probability from Monte Carlo simulations of subglacial routing indicates the highest-probability catchments include the lower portion of KIS, but lower-probability catchments (<35% of simulations) extend into the upper Kamb and cross into the upper regions of Whillans Ice Stream (WIS). Three documented subglacial lakes (blue dots labelled KT1, KT2, KT3) are present in the lower Kamb trunk catchment, and an additional 13 lakes (blue dots) lie within the 10% probability contour. The subglacial access borehole (red triangle) is located at the downstream end of KIS’s subglacial network. Locations of provenance observations (*ε*_Nd_) from Whillans Ice Stream (*ε*_WIS_), upstream on KIS (*ε*_KIS_), Bindschadler Ice Stream (*ε*_BIS_), Whillans Grounding Zone (*ε*_WGZ_) and the ice-shelf sites *ε*_J9_ and *ε*_HWD2_ are shown as coloured boxes (Methods and Supplementary Table 2). **b**, The grounding-zone channel^34^ is geographically continuous with reduced ice thickness downstream and prominent surface features visible in Moderate Resolution Imaging Spectroradiometer (MODIS) Mosaic of Antarctica (MOA)^50^. Grounding-zone channel and region of reduced ice thickness shown by red arrows. The portion of the grounding line used to estimate modelled subglacial flux is shown by the orange line. In both **a** and **b**, the grounding-zone transition from grounded ice to floating ice shelf is shown by the black line^35^. Source data

Below 25 cm, unit 4 (25-40 cm) ranges from poorly sorted at depth to moderately sorted (Fig. 4). Geological provenance proxies from unit 4 (*ε*_Nd_ and ^87^Sr/^86^Sr; Fig. 4c) indicate a different source geology when compared with the other units. Unit 4’s provenance is most similar to samples taken from upstream and downstream on KIS and the adjacent Whillans Ice Stream^41^ (Figs. 4c and 5). The poor sorting and lack of preferred clast orientation in unit 4 indicate it has not settled through a water column and is unlikely to be the direct result of the melt-out of basal debris. Given this, and the sharp basal contact, we suggest this unit resulted from a density flow related to a discharge event^40^.

**Extended Data Fig. 4:**
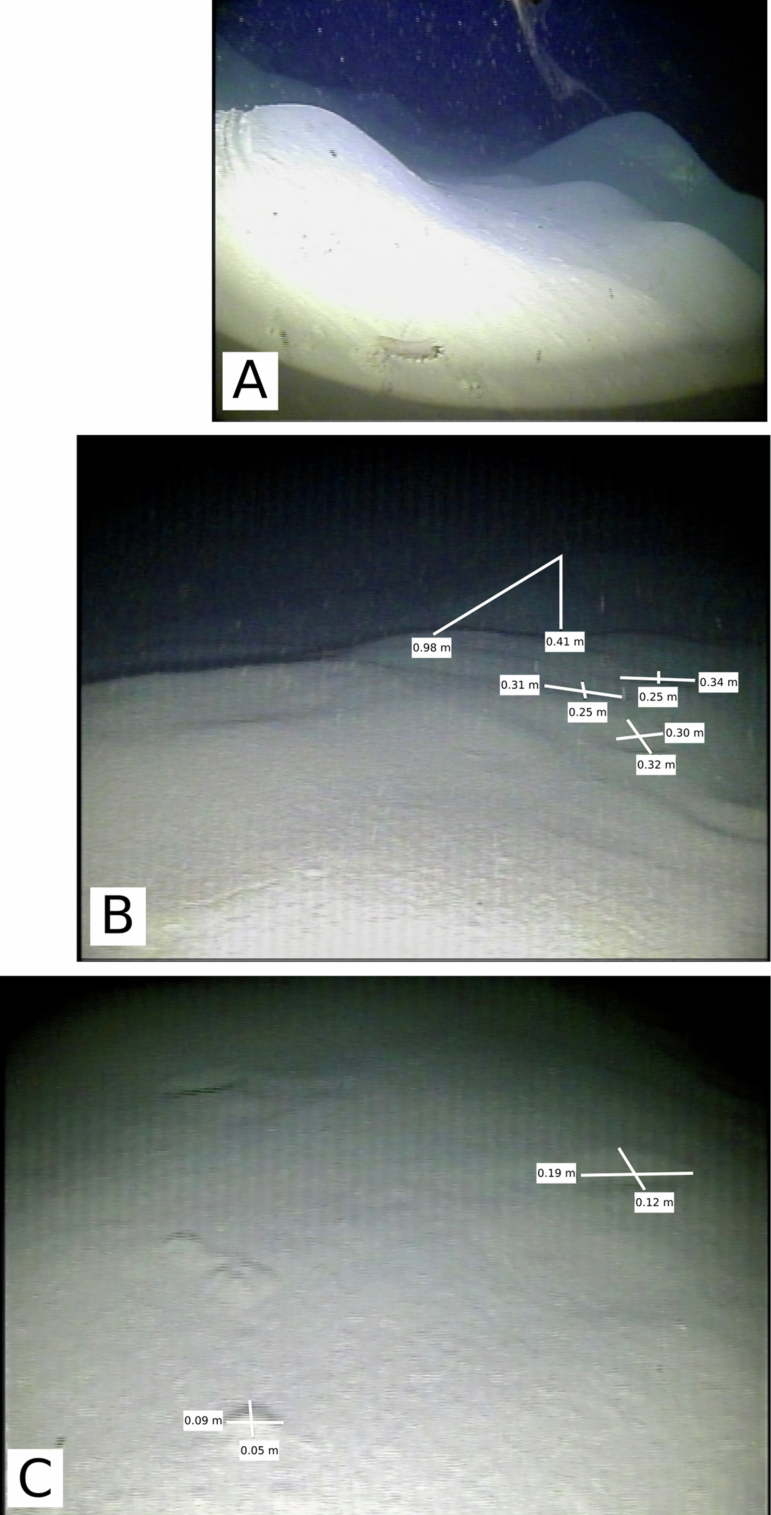
Channel floor imagery. Imagery from beneath the borehole obtained with two Deepsea Nano SeaCam standard definition video cameras mounted on the front of the Remotely Operated Vehicle (ROV) Icefin^4,6^. The scale of features in the images was measured with an adjacent Blueprint Subsea Oculus multibeam imaging sonar onboard Icefin following the methodology reported in^4,6^. **A**) Seafloor image with no scaling information available. **B**), **C**) Seafloor images with annotated scaling. Source data

The lowermost unit (5) consists of Miocene-age diatom clasts, many of which are intact, in a diatom-rich matrix, indicating this deposit is not the result of a discharge event. Miocene diatom deposits are found throughout this sector of Antarctica, and their biostratigraphic age is generally not considered to represent their age of deposition^4,42^. Imagery from the channel floor within 350 m of the borehole (Extended Data Fig. 4) shows decimetre- to metre-scale boulders. Fluvial transport of these boulders would require fluxes one to two orders of magnitude higher than we observe^43^. It is possible that this lowest unit sampled either an in situ deposit or a boulder of unknown origin.

The likely subglacial discharge origin and geological provenance of units 1–4 indicate subglacial discharge variability. Material transported from different regions of the ice-stream bed has crossed the same location at the grounding zone at different times and been preserved in the sediment record. The depositional mode records the last phase of transport, and we note that the distinct provenance of unit 4 may result from the melt-out of basal debris during a discharge event that was then remobilized and represents the source geology at the site of basal accretion. These findings have implications for the interpretation of ice-sheet proximal sedimentary sequences (for example, ref. ^44^) as provenance changes may occur even when ice flow remains largely unchanged.

## Upstream catchment and downstream impacts

**Extended Data Fig. 5:**
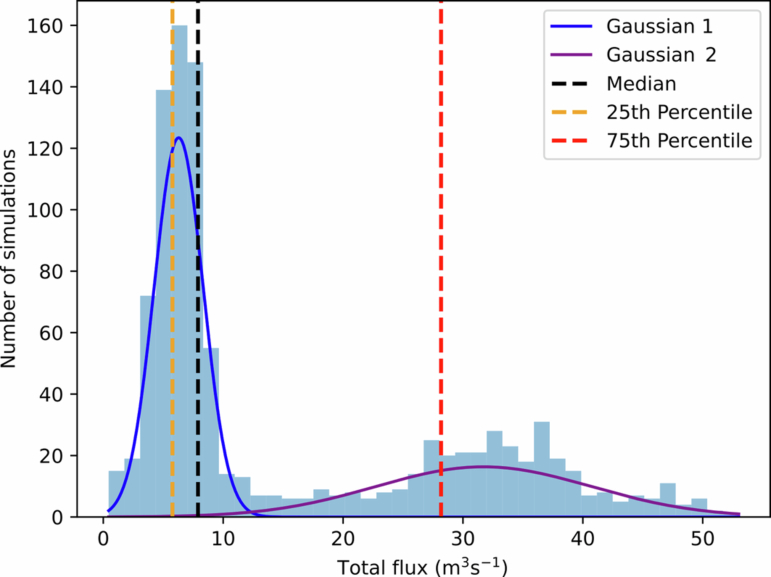
Subglacial flux distribution. Histogram showing the distribution of total subglacial flux estimates from 1000 simulations of basal routing. Median, first quartile and third quartile values for the entire distribution are shown by dashed vertical lines. The resulting Gaussian distributions from a two-member Gaussian mixing model are shown by solid blue and purple lines. For flux gate location and catchment area see manuscript Fig. 4b. Source data

The source of subglacial discharge upstream of the channel can be constrained using probabilistic estimates of subglacial routing^45^ (Fig. 5a). These hydrological catchments are distinct from the more commonly used ice-flow catchments and indicate the source regions of subglacial water and fluvial sedimentary deposits originating beneath ice sheets. The most likely catchment for subglacial water emerging at our channel site encompasses 22,000 km^2^ on the lower KIS (90% probability; Fig. 5a). Lower-probability catchments encompass much of the upper KIS (Fig. 5a). Combining these routing and catchment estimates with melt estimates^46^ allows us to model subglacial water flux across our borehole site. The median modelled discharge is 7.9 m^3^ s^−1^ (5.8–28.2 m^3^ s^−1^, 25th–75th percentiles; Extended Data Fig. 5), and more than 99% of our catchment estimates result in subglacial discharge greater than the discharge we estimate using oceanographic observations. Part of the mismatch between modelled and observed discharge may be due to a more distributed subglacial network than suggested by the routing^45^. However, the concentration of flow routing beneath KIS (Fig. 1) and the absence of ice-shelf features indicating melt elsewhere suggest our channel observations are unlikely to be missing much discharge.

**Extended Data Fig. 6:**
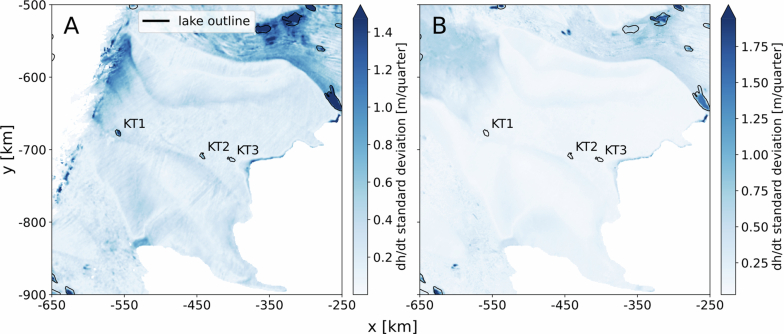
Subglacial lake activity. Active subglacial lake activity identified using the standard deviation of gridded height-change rates (dh/dt) for the trunk of Kamb Ice Stream. **A**) Standard deviation of dh/dt estimated over three-month intervals from CryoSat-2 elevation observations between 2010-07-02 and 2018-09-30 (N = 34). **B**) Standard deviation of dh/dt estimated over three-month intervals from ICESat-2 elevation observations between 2018-10-01 and 2023-04-02 (N = 19). Black polygons labelled KT1, KT2, and KT3 denote subglacial lake outlines^8^. Colormap is capped at the 97th percentile of standard deviation to highlight outliers. Source data

**Extended Data Fig. 7:**
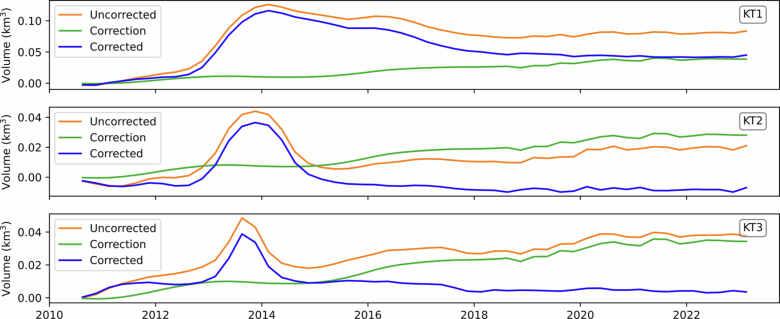
Subglacial lake timeseries. Estimated time series of subglacial lake volume change for active subglacial lakes KT1, KT2, and KT3. Uncorrected curves represent the spatially averaged dh/dt within subglacial lake outlines of KT1-KT3 multiplied by the area of each outline. Correction curve represents the off-lake area extending 10 km from the lake outline. Corrected time series is the uncorrected minus correction time series, which represents the inferred subglacial lake volume change. Source data

The extent of the subglacial catchment also determines the active subglacial lakes that can contribute to the time-varying component of subglacial hydrology. Three documented active subglacial lakes^8,36^ (KT1–KT3, Extended Data Figs. 6 and 7) are present in the lower KIS catchment, and an additional 13 active subglacial lakes^7^ lie within the 10% catchment probability contour (Fig. 5a). The active subglacial lakes in the lower KIS catchment have exhibited one fill–drain cycle within the satellite observation period (2003–present) (Extended Data Fig. 7).

**Extended Data Fig. 8:**
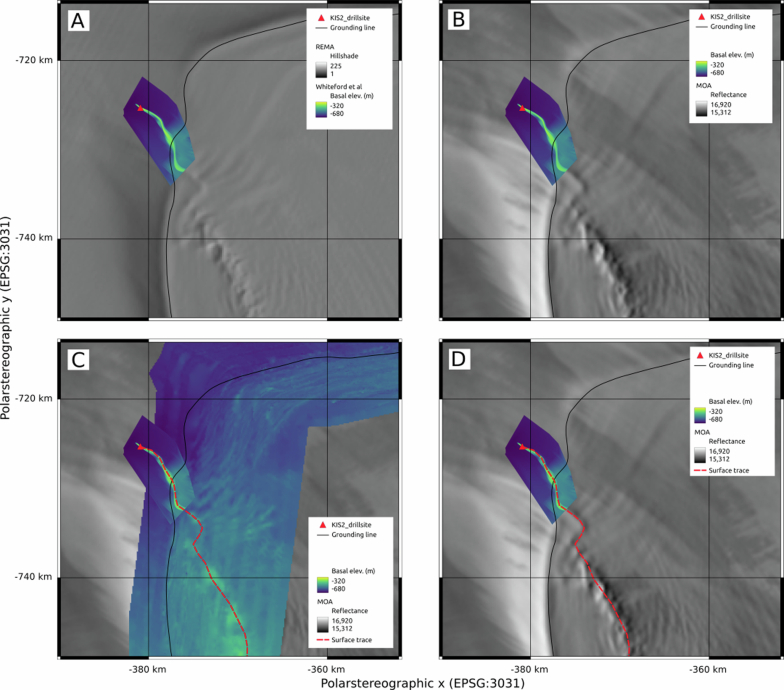
Continuation of channel downstream. Surface imagery and ice thickness observations downstream of the borehole location (red triangle shows). **A**) Grounding zone channel bed elevation^8^ overlain on REMA surface elevation hillshade^20^. **B**) Grounding zone channel bed elevation^34^ overlain on MODIS MOA reflectance^50^. **C**) Grounding zone channel^34^ and swath radar imaged bed elevation overlain on MODIS MOA reflectance^50^. Red dashed line denotes suspected plume pathway. **D**) Grounding zone channel^34^ and suspected plume pathway (red dashed line) overlain on MODIS MOA reflectance^50^. Source data

Downstream of the grounding-zone channel, notable surface features are apparent in satellite imagery^14^ (Fig. 5b and Extended Data Fig. 8) and surface elevation models^47^. In places, these features cut across indicators of past flow (streak lines), indicating that they developed since the stagnation of the ice stream approximately 190 years ago^30,37^. Ice thickness observations from swath-processed airborne radar show these surface features correlate with regions of thinner ice (Fig. 5b). Both the surface features and the associated regions of thin ice are geographically continuous with the grounding-zone channel (Extended Data Fig. 8). The region’s history is complex. Before stagnation, it was an active shear margin, and after stagnation the grounding zone retreated, most likely in a stepwise manner^37^. Weakening and thinning within the shear margin would make the ice shelf more vulnerable to subglacial drainage-sourced basal melt^48^, and stepwise retreat would allow melt features to amplify. Plume theory indicates subglacial discharge events amplify melt but also decrease in impact with distance from their source^11^. Given the history of this part of the ice shelf, we suggest a progressive development of ice-shelf features in response to episodic subglacial drainage-induced melt exploiting the former shear margin, and grounding-zone retreat increasing the distance over which these impacts occurred.

## Episodic discharge and ice-shelf evolution

We directly observed subglacial water emerging from beneath the West Antarctic Ice Sheet and interacting with cavity water beneath the Ross Ice Shelf. The observed subglacial discharge flux of 0.9 ± 0.4 m^3^ s^−1^ is smaller than the modelled long-term estimate of 7.9 m^3^ s^−1^. Much of this difference can be explained by episodic discharge events, whereby subglacial water is stored and released by the catchment upstream^8,36^ (Extended Data Figs. 6 and 7). Sediment deposits within the channel interpreted to result from previous discharge events have sampled distinct geological provenances, indicating the subglacial hydrologic system may be capable of spatial variability (Figs. 4 and 5a). Sediment deposits related to subglacial discharge probably all postdate the migration of the channel over this location sometime since 1985^34^, and the most recent large event (Unit 2; Fig. 4) probably resulted from the subglacial lake drainage event observed in the satellite record (Extended Data Fig. 7).

**Extended Data Fig. 9:**
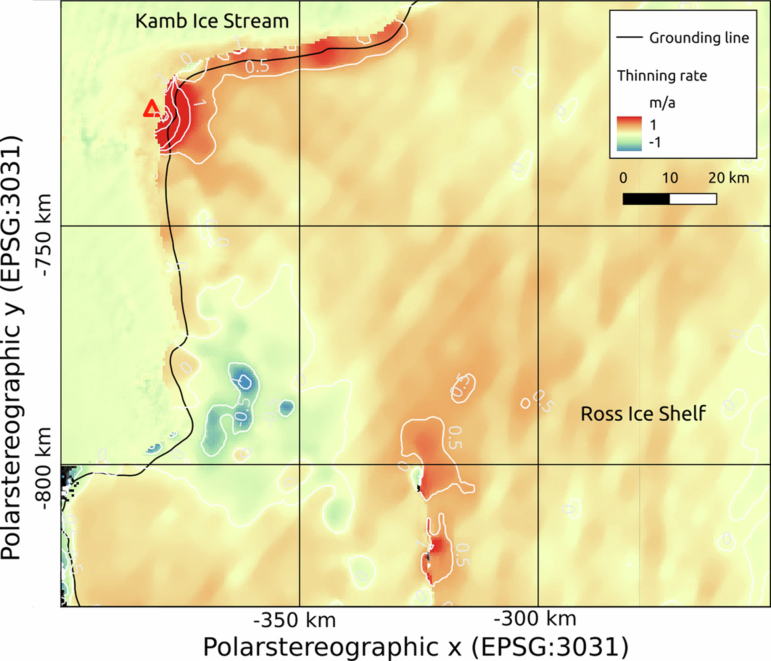
Ice shelf basal mass balance and ice sheet surface elevation change. Ice shelf basal mass balance estimated using CryoSat-2 radar altimetry [Previously published data from:^49^] and onshore surface elevation change estimated in 500 m tiles using elevation data generated using swath processed CryoSat-2 data^34^ following the method of^27^. Contours on the ice shelf at 0.5 m a^−1^ intervals. Borehole access location shown by red triangle. Grounding line shown as black line^35^. Source data

Downstream, the impact of subglacial discharge is manifest in the record of melt left behind in the ice-shelf base and manifest on the surface^34^ (Fig. 5b and Supplementary Figs. 1 and 2). The subglacial discharge plume we observed was not in contact with the roof of the channel (Fig. 3). With greater discharge flux and associated enhanced buoyancy, mixing and entrainment, the discharge plume would reach the channel apex with greater melt potential^11^, would influence a larger region of the ice shelf^19^ and may have contributed to the formation of the existing keyhole-like shape of the grounding-zone channel. The increased turbulence provided by episodic discharge events has the potential to overcome stratification in the ocean-cavity water column that otherwise can reduce the basal melt of ice shelves^18,24^. The resulting distributed melt would help explain the regional thinning observed^49^ (Extended Data Fig. 9). Episodically high melt rates would also further reduce the restorative role of internal ice flow^23^ and result in further mechanical weakening^21,22,48^.

The implications of subglacial discharge for ice-shelf melt and sea-level projections have begun to be explored^11,16,19^. Our findings emphasize the importance of episodic subglacial discharge, which is likely to amplify the impact that subglacial water has on ice shelves and ice sheets.

## Methods

### Borehole access

The borehole was drilled at 82.47048° S, 152.29145° W using hot water, heated on the surface and expelled through a hose ending in a lance tipped with a high-pressure nozzle^51^. Borehole science was conducted between 28 December 2021 and 13 January 2022. The borehole was initially reamed to a nominal diameter of 0.3 m. A blockage of the borehole on 31 December 2021 led to repositioning of the borehole approximately 10 m downstream to 82.47040° S, 152.29192° W, and borehole science resumed on 3 January 2022. Borehole operations cycled between science operations interspersed by reaming of the borehole, which occupied approximately 6 hours every 24 hours. Borehole access timing and modelled tidal elevation^52^ are shown in Extended Data Fig. 1.

### Channel imaging and velocity profiling

Profiling with a rotating 500 kHz altimeter (Subsea ISA500) allowed us to image the roof, walls and floor of the grounding-zone channel. The altimeter was lowered through the borehole and water column tilted at various angles from the vertical. During profiling, the line was rotated slowly. Individual range estimates have sub-centimetre accuracy. A best-fitting cross section of the channel walls was estimated by spline-fitting a polygon to the range estimates. A region of uncertainty in the lower right-hand corner was included due to a lack of altimeter returns from this region. This uncertainty does not effect our flux and melt estimates due to the low fractions of subglacial discharge water and glacial meltwater in the bottom portion of the channel.

During velocity profiling, a Nortek Aquadop current meter measured water-mass velocity and bearing, and an RBR Duet recorded temperature and pressure. The Aquadop had an averaging interval of 1 s and a sampling interval of 1 s. The current observations in the top 50 m of the channel were affected by weak backscatter and noise from the Subsea altimeter, leading to greater apparent variability in this region. At the end of the survey (13 January 2022), we deployed a mooring with Nortek Aquadop Deep Water Current meters at heights of 9, 162 and 222 m above the channel floor. These sampled at a 20 s averaging interval with a sampling interval of 30 minutes. As with the profiling data, the upper mooring location was affected by weak backscatter and contamination from the altimeter, so was omitted from our mooring–profiling comparison.

### Hydrographic profiling

The water-column structure was measured by lowering an RBR Concerto CTD (conductivity, temperature, pressure and turbidity) package. Data processing included correcting the pressure record for atmospheric and tidal effects, removing erroneous observations caused by instrument equilibration and icing, and combining remaining casts in each session into a single mean profile binned at 1 m vertical intervals. Aquadop pressure, current velocity and temperature records were treated similarly but binned at 2 m intervals to account for fewer observations.

Practical salinity and in situ temperature were converted to absolute salinity (*S*_A_) and conservative temperature (*Θ*) following TEOS-10 (Thermodynamic Equation of Seawater 2010)^53^. Turbidity profiles were calibrated using paired observations from out sensor with that reported previously^29^. The average turbidity within the top 40 m of the channel for each profile was then subtracted to aid comparison between casts. The average within the top 40 m from all profiles was then added to preserve the absolute value.

### Water-mass partitioning and flux estimation

Water masses were partitioned using temperature and salinity and a three-member mixing model following refs. ^13,54,55^. The source water was assumed to be HSSW and to have the properties of the deepest water body we observe. We note that our HSSW source water (Extended Data Table 1) is approximately 0.05 °C warmer and approximately 0.04 g kg^−1^ fresher than HSSW observed at the ice front^55^ and in the ocean cavity approximately 55 km away^29^. This suggests that the HSSW we observe retains some signature of modified circumpolar deep water. This observation does not affect our analysis, but it is of interest considering the distance from the ice front (approximately 470 km) and the absence of modified circumpolar deep water nearby.

We assumed the HSSW was well mixed under fully turbulent conditions with ice-shelf GMW and SGW. The effective conservative temperature of GMW (*Θ*_GMW_) was estimated as –92.5 °C following ref. ^13^, and the conservative temperature of SGW (*Θ*_SGW_ = –0.48 °C) was estimated from the melting point of ice at 650 m depth, which is the approximate depth of the point where the subglacial channel meets the grounding-zone channel^34^.

Assuming the water column was well mixed and consisted of three known water masses (Supplementary Table 1) allowed us to estimate the proportions of each water mass (*p*_1_, *p*_2_, *p*_3_) from the observed values of *Θ* and *S*_A_ in the following way:

$$\begin{array}{c}{p}_{1}{\Theta }_{1}+{p}_{2}{\Theta }_{2}+{p}_{3}{\Theta }_{3}=\Theta \\ {p}_{1}{S}_{\rm{{A}}_{1}}+{p}_{2}{S}_{\rm{{A}}_{2}}+{p}_{3}{S}_{\rm{{A}}_{3}}={S}_{{\rm{A}}}\\ {p}_{1}+{p}_{2}+{p}_{3}=1\\ A=\left[\begin{array}{ccc}{\Theta }_{1}&{\Theta }_{2}&{\Theta }_{3}\\ {S}_{\rm{{A}}_{1}}&{S}_{\rm{{A}}_{2}}&{S}_{\rm{{A}}_{3}}\\ 1&1&1\end{array}\right]\\ B=\left[\begin{array}{c}\Theta \\ {S}_{{\rm{A}}}\\ 1\end{array}\right]\\ x=\left[\begin{array}{c}{p}_{1}\\ {p}_{2}\\ {p}_{3}\end{array}\right]\end{array}$$

We estimated water-mass proportions (*x* = *A*^−1^*B*) from average values of *Θ* and *S*_A_ in 1 m vertical bins for each of the six profiles presented in Fig. 3e,f. *Θ* and *S*_A_ observations were obtained during six independent casts between 28 December 2021 and 1 January 2022. (See Extended Data Fig. 1 for experiment timing in relation to tidal state.)

To convert these water-mass proportions to flux estimates, we combined the mass-proportion estimates with independent water-column velocity estimates (Fig. 3b,c). Water-column velocity was observed during six casts (three up casts and three down casts) on 9 January 2022. The water-column velocity observations included temperature but not salinity. To allow for variability in the vertical position of the plume, we combined the observations of velocity and temperature, and temperature and salinity, on the basis of temperature. To accomplish this, we divided the water column into four distinct layers: the upper layer, the plume layer, the lower layer and the bottom layer. For layer definitions, see Supplementary Table 2 and Extended Data Fig. 2.

Layer water-mass fractions and velocities were then estimated using area weighting per bin to account for the varying channel width with depth. To account for the correlation between layer areas and mass fractions, the equivalent areas of the water-mass fractions per layer were used to estimate fluxes. The standard deviations of layer properties were used to obtain an estimate of the variability in flux during the experiment. Summary results are presented in Supplementary Tables 3–5. Our methods quantify the variability observed during our observation period and probably underestimate variability outside of our times of observation. Additional unquantified uncertainty comes from our assumption of uniform cross-channel properties implicit in our use of cross-channel area in our flux estimates.

### Coring

A 0.53-m-long sediment core was recovered using a gravity corer deployed though the borehole. The corer was weighted and fitted with a sterilized 52 mm (inner diameter) polycarbonate core tube. The corer was fitted with a core catcher and lowered through the borehole and allowed to freefall from approximately 20 m above the seafloor. After recovery, the water from the core headspace was drained, and the core was packed with porous foam. The core was then sealed and stored horizontally at 4 °C. The core was CT scanned at a voxel size of 0.2734, 0.2734, 0.625 mm (*x*, *y*, *z*) using a GE Medical Systems Brightspeed CT scanner. Densities measured in Houndsfeld units were converted to density in kg m^−3^ following ref. ^56^. A representative density profile was estimated from the average of the central 10 × 10 voxels in each vertical slice. After CT scanning, the core was split lengthwise with a Geotek core splitter. The split core was then photographed and scanned using a hyper-spectral scanner with a spatial resolution of approximately 0.4 mm.

#### Grain-size analysis

Sediment samples were collected and treated with 27% hydrogen peroxide (H_2_O_2_) to remove organic material by digestion until completion of the reaction. Samples were topped up with deionized water and centrifuged three times before being decanted into glass beakers, and the surfactant Calgon (1 g l^–1^ Na_6_O_18_P_6_) was added to ensure particles remained disconnected and in suspension. Samples were sonicated in an ultrasonic bath for 20 minutes with stirrers and analysed with the Malvern Laser Sizer 3000 using the aqueous module. Samples with larger grain sizes were sieved at 1,400 μm, the practical upper limit of the Malvern Laser Sizer.

#### Nd and Sr isotopes

Sediment samples were prepared for Nd and Sr isotope measurements as previously described^44^^,^^57^. The <63 μm fraction was leached to remove authigenic coatings, and the Nd and Sr were isolated using established ion exchange chromatography methods. Neodymium isotopes were measured in the MAGIC laboratories at Imperial College London on a Nu high-resolution multi-collector inductively coupled plasma mass spectrometer. To account for instrumental mass bias, isotope ratios were corrected using an exponential law and a ^146^Nd/^144^Nd ratio of 0.7219. Interference of ^144^Sm on ^144^Nd, although negligible, was corrected for. To correct measured ^143^Nd/^144^Nd ratios to the commonly used JNdi-1 value of 0.512115 (ref. ^58^), bracketing standards were used. USGS (US Geological Survey) BCR-2 rock standard measurements yielded ^143^Nd/^144^Nd ratios of 0.512639 ± 0.000002 (*n* = 3), in very close agreement with the published ratio of 0.512638 ± 0.000015 (ref. ^59^). A full procedural blank was 25 pg of Nd. Final ^143^Nd/^144^Nd ratios are expressed using varepsilon notation (*ε*_Nd_), which denotes the deviation of a measured ratio from the modern chondritic uniform reservoir (0.512638) in parts per 10,000 (ref. ^60^).

Strontium isotopes were measured in the MAGIC laboratories at Imperial College London on a Triton thermal ionization mass spectrometer. Samples were loaded in 1 μl of 6 M HCl onto degassed tungsten filaments with 1 μl of TaCl_5_ activator. The measured ^87^Sr/^86^Sr ratios were corrected for instrumental mass bias using an exponential law and an ^88^Sr/^86^Sr ratio of 8.375. Interference of ^87^Rb was corrected for using an ^87^Rb/^85^Rb ratio of 0.386. Samples were corrected to the published NIST (National Institute of Standards and Technology) 987 standard reference material value of 0.710252 ± 0.000013 (ref. ^59^). These were completed every four unknowns, with a mean of 0.710246 ± 0.000013 (2 s.d., *n* = 19). Accuracy of results was confirmed using rock standard USGS BCR-2, processed with every batch of samples, which yielded ^87^Sr/^86^Sr ratios of 0.705012 ± 0.00017 (2 s.d., *n* = 10). This is in agreement with the published ratio of 0.705013 ± 0.00010 (ref. ^59^). *ε*_Nd_ values plotted in Fig. 4a are shown in Supplementary Table 6.

#### Diatom age determination

The lowermost sedimentary unit (Unit 5) was determined to be Miocene in age on the basis of the diatom assemblage reported previously^61^. Diagnostic taxa placed the unit in the *Thalassiosira praefraga* Range Zone with overlap between *T. nansenii* and *T. praefraga* suggesting the unit was sourced from deposits of 18.7–18.0 Ma age^42,61,62^.

### Subglacial routing and catchment modelling

Subglacial routing and catchments were determined using hydropotential gradients^6^ and followed the stochastic D8 method described previously^45^. This allowed probability to be quantified using a Monte Carlo ensemble with 1,000 runs that sampled Gaussian random fields to make realizations of surface elevation, bed elevation, flotation fraction (the ratio of subglacial water pressure to ice overburden pressure) and subglacial melt input. Surface elevation and bed elevation were obtained from REMA (200 m spatial resolution)^47^ and BedMachine (500 m spatial resolution)^63^, respectively. An average flotation fraction of 1.0 was assumed. The subglacial melt input is from ref. ^46^. The Gaussian random fields used correlation lengths of 10 km for the bed elevation, surface elevation and flotation fraction and a correlation length of 20 km for the subglacial melt. The amplitude of the bed elevation’s Gaussian random field, which is expressed as a standard deviation (*σ*), uses the spatially varying error field provided in the BedMachine dataset (average value of 93 m within the KIS catchment); similarly, the *σ* for the subglacial melt uses the standard deviation field provided in that dataset. The surface elevation *σ* was set to 0.6 m (ref. ^47^) plus 10% of the firn thickness^63^, and the flotation fraction *σ* was set to 0.03. Subglacial catchments were estimated by determining the flow paths upstream of an approximately 18-km-long portion of the KIS grounding zone (Fig. 4a). Subglacial flux was estimated similarly as the flux crossing that stretch of the grounding line (Fig. 4b and Extended Data Fig. 5). A mismatch between modelled subglacial routing and the actual channel location of approximately 3.5 km was observed. This mismatch was determined to be a model artefact due to the absence of any other grounding-zone channel away from the main channel as determined by oversnow radio echo sounding^34^.

### Subglacial lake activity

We estimated subglacial lake activity using CryoSat-2 and ICESat-2 radar and laser altimetry data. We grid-phase unwrapped Synthetic Aperture Radar-Interferometric mode CryoSat-2 radar altimetry data (following ref. ^2^) for the period July 2010 to the start of ICESat-2 era (October 2018) mimicking ICESat-2’s ATL15 data product’s spatial structure and time periods (propagating time periods back at 3-month intervals to the start of the CryoSat-2 mission). During the ICESat-2 era, we used a higher-level ICESat-2 data product, ATL15 Gridded Antarctic Land Ice Height Change r003, that provides gridded estimates of ice-sheet elevation change across Antarctica at a spatial resolution of 1 km and a temporal resolution of 91 days from during the ICESat-2 era^64^ (October 2018 to April 2023). We used the standard deviation across a total of 53 time steps of gridded ice-surface height-change rates estimated over 3-month periods to highlight areas of inferred subglacial lake activity (Extended Data Fig. 6). According to the gridded standard deviation estimates for each mission, subglacial lakes KT1, KT2 and KT3 were active between 2 July 2010 and 30 September 2018 (Extended Data Fig. 6a) and inactive between 1 October 2018 and 2 April 2023 (Extended Data Fig. 6b).

We estimated volume change time series (following ref. ^8^) by calculating the mean change in surface elevation with time (d*h*/d*t*) within each published subglacial lake outline at quarterly (3-month) intervals (Extended Data Fig. 7). We corrected this height-change time series for regional secular height change by subtracting the mean height change in an area surrounding the lake extending 10 km from the lake outline for each time step (following ref. ^65^). We then integrated the d*h*/d*t* time series through time and multiplied the corrected time series by the area of each outline to estimate surface ice-volume displacement. This approach assumes a 1/1 ratio of ice volume to water volume displacement (for example, refs. ^66–68^); although this approach is commonly implemented, the 1/1 assumption may be an oversimplification in slow-flowing, highly viscous ice settings, such as the KIS trunk^69^.

### Swath radar imaging

Swath radar data were collected between 20 December 2013 and 27 December 2013 using the Center for Remote Sensing and Integrated Systems eight-element array of the Multichannel Coherent Radar Depth Sounder radar system^70^. This swath imaging radar was installed on a Basler air frame that flew at approximately 1.8 km above the ice surface. Processing followed refs. ^71,72^ and involved digitizing the basal return in cross-track images. This resulted in swath widths of approximately 1 km in our study area, with nominal along-track sampling every 15 m and across-track sampling every 70 m (Fig. 4b).

## Online content

Any methods, additional references, Nature Portfolio reporting summaries, source data, extended data, supplementary information, acknowledgements, peer review information; details of author contributions and competing interests; and statements of data and code availability are available at 10.1038/s41561-025-01687-3.

## Supplementary information

Supplementary Information Supplementary Figs. 1 and 2, Tables 1–7 and Discussion.

## Source data

Source Data Fig. 1 Raster of flux estimates (flux_masked.tif).

Source Data Fig. 2 Channel-wall local coordinates (kis2channelxyz.csv), velocity profiling oceanographic observations (kis2velocityprofiling.csv), turbidity cast (kis2turcast8.csv) salinity cast (kis2salcast8.csv) and temperature cast (kis2temcast8.csv).

Source Data Fig. 3 .csv files of all velocity profiling and temperature, salinity and turbidity casts. .csv files of water-mass estimates.

Source Data Fig. 4 Image of sediment core and CT scan (KIS2Sed_nolabels_v3.tif), provenance data (kis2provenance.csv), grain-size data (kis2Grainsize.csv) and density data (kis2CTdensity.csv).

Source Data Fig. 5 Hydrological catchment probabilities (catch_channel3_b31e69d7.tif), spatial provenance data (kis2provenanceSpatial.csv) and swath radar data (kis2_swath_masked.tif).

Source Data Extended Data Fig. 1 .txt file containing time stamp and tidal elevation.

Source Data Extended Data Fig. 2 .csv files containing temperature during velocity profiling (kis2velocityprofiling.csv) and temperature–salinity casts (kis2temcast?.csv).

Source Data Extended Data Fig. 3 Grain-size distribution.

Source Data Extended Data Fig. 4 Channel-floor imagery of dropstones.

Source Data Extended Data Fig. 5 Model estimates of flux across grounding zone.

Source Data Extended Data Fig. 6 Gridded standard deviation of elevation-change rates (d*h* /d*t*) for the trunk of Kamb Ice Stream estimated using CryoSat-2 and ICESat-2 elevation observations.

Source Data Extended Data Fig. 7 Time series of subglacial lake volume change.

Source Data Extended Data Fig. 8 Raster of ice elevation of subglacial channel (highres_ice_base_line2line.tif) and swath-processed ice-shelf thickness (kis2_swath_masked.tif)

Source Data Extended Data Fig. 9 Surface elevation change for grounded KIS.

## Figures and Tables

**Extended Data Table 1:**
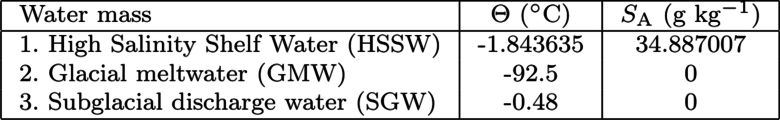
Water column properties

Conservative temperature (*Θ*) and absolute salinity (*S*_A_) for the three water sources used in water mass partitioning.

## Data Availability

Data presented in this study are available via Zenodo (10.5281/zenodo.14942664). MODIS MOA imagery is available at https://nsidc.org/data/nsidc-0593/versions/2. CryoSat-2 swath-processed elevation data are available at https://cryotempo-eolis.org/ (ref. ^73^) and 10.5281/zenodo.14963550. ICESat-2 ATL15 data are available at 10.5067/ATLAS/ATL15.004. REMA elevation data are available at https://www.pgc.umn.edu/data/rema/. Source data are provided with this paper.

## Code availability

Data processing and plotting were performed using Python. Geospatial data were plotted using GMT^74^ and Quantarctica^75^.
