# Supplementary material for: A West Antarctic grounding-zone environment shaped by episodic water flow
Source: Nat Geosci. 2025 May 12;18(5):389–95. doi: 10.1038/s41561-025-01687-3 (PMC12075004; doi:10.1038/s41561-025-01687-3)
Supplement: Supplementary file 1 — Supplementary Figs. 1 and 2, Tables 1–7 and Discussion. [file 41561_2025_1687_MOESM1_ESM.pdf]

nature geoscience

Supplementary information https://doi.org/10.1038/s41561-025-01687-3

A West Antarctic grounding-zone environment shaped by episodic water flow

In the format provided by the authors and unedited

Water mass partitioning

Water mass partitioning used the properties shown in Supplementary Table 1 and the layer definitions in Supplementary Table 2. The resulting layer and water mass areas from temperature and salinity profiling are shown in Supplementary Table 3 and the layer area and velocity estimates from velocity profiling are shown in Supplementary Table 4. A summary of flux estimates is presented in Supplementary Table 5.

Water mass	Θ (°C)	S_A (g kg^{-1})
1. High Salinity Shelf Water (HSSW)	-1.843635	34.887007
2. Glacial meltwater (GMW)	-92.5	0
3. Subglacial discharge water (SGW)	-0.48	0

Supplementary Table 1 Conservative temperature (Θ) and absolute salinity (S_A) for the three water sources used in water mass partitioning.

**Layer**	**Top definition**	**Bottom definition**
**Upper layer**	$z_{\max}$	z at top of plume
**Plume layer**	z at first $\mathrm{T}(z) > \overline{\mathrm{T}}_{\mathrm{top\ 10\ m}} + 0.005\,°\mathrm{C}$	z at $\min_{z>156\,\mathrm{m}} \mathrm{T}(z)$
**Lower layer**	z at bottom of plume layer	z at top of bottom layer
**Bottom layer**	z at first $\mathrm{T} < \mathrm{T}_{\max} - 0.01\,°\mathrm{C}$	$z_{\min}$

Supplementary Table 2 Temperature (T) based layer boundary (z) definitions used to combine water column velocity and temperature–salinity observations in order to estimate subglacial discharge water flux. The resulting layers are shown in Extended Data Figure E2.

	**Total Area (m^2)**	**SGW Area (m^2)**	**GMW Area (m^2)**
**Upper**	3788 (880)	20.8 (4.2)	14.5 (3.4)
**Plume**	8636 (1123)	37.3 (8.7)	16.6 (3.9)
**Lower**	15325 (966)	0.9 (2.2)	8.5 (0.9)
**Bottom**	1803 (145)	0.04 (0.26)	0.07 (0.04)

Supplementary Table 3 Layer area statistics during temperature and salinity profiling. Bracketed values denote standard deviation of estimates. SGW and GMW denote the effective areas of subglacial discharge water and glacial melt water respectively.

	**Total Area (m^2)**	**Velocity ($m\ s^{-1}$)**
**Upper**	3566 (514)	-0.0019 (0.006)
**Plume**	9717 (642)	0.026 (0.008)
**Lower**	13982 (359	-0.0067 (0.0039)
**Bottom**	2124 (176)	-0.0250 (0.0031)

Supplementary Table 4 Layer area and velocity statistics during velocity profiling. Bracketed values denote standard deviation of estimates. Positive velocities represent down-channel flow.

	**Q (m^3s^{-1})**	**SGW Q (m^3s^{-1})**	**GMW Q (m^3s^{-1})**
**Upper**	-7.2 (21.9)	-0.04 (0.12)	-0.03 (0.08)
**Plume**	224.9 (71.6)	0.97 (0.37)	0.43 (0.16)
**Lower**	-102.6 (60.9)	-0.0058 (0.0018)	-0.057 (0.034)
**Bottom**	-45.1 (6.7)	0.0009 (0.0067)	-0.0019 (0.0010)
**Total**	70.0 (97)	0.93 (0.39)	0.35 (0.19)

Supplementary Table 5 Flux estimates per layer including total flux (Q), subglacial discharge water (SGW Q), and glacial melt water (GMW Q). Standard deviation shown in brackets.

Site	Latitude	Longitude	ε_{Nd}
WIS*	-83.45	-137.76	-8.43
KIS*	-82.43	-135.95	-7.31
BIS*	-81.07	-140.01	-9.93
J9	-82.38	-168.63	-7.27
HWD2	-80.66	174.46	-10.91
WGZ	-84.34	-163.61	-8.74

Supplementary Table 6 ε_{Nd} values and locations shown in Article Figure 4A. Sites marked with * are from [1].

Provenance data and grain size distribution

Provenance data from Article Figure 4A are shown in Supplementary Table 6. Complete grain size distributions for the <1400 μm fraction for all samples processed from the KIS2 sediment core (Article Figure 3D) are shown in Extended Data Figure E3. The grain size distribution in unit 1 (0–6 cm) includes an approximately 10 μm fraction observed in meltwater deposits on the Ross Sea continental shelf [2], alongside coarser fractions. Units 2 and 3 (6–25 cm) exhibit grain size distributions with similar sand modes to samples from beneath the adjacent Whillans Ice Stream [3, 4], and coarser than those interpreted as derived from subglacial water in more distal cores

[2, 5]. Unit 4 exhibits a sharp basal contact, and large (cm-scale) clasts with no apparent preferred orientation. While all units exhibited biogenic content, unit 4 had the least [<3 %, compared to >20 % in all other units, 6].

Surface elevation and thickness change

Other observations suggest episodic discharge events punctuate the low background subglacial drainage we observed. Surface elevation change was estimated using a geodetic-grade Global Navigation Satellite System (GNSS; Septentrio AsteRx-m UAS unit and Septentrio PolaNt-x MC antenna) mounted on an aluminium pole logging at 30 second intervals. This GNSS unit was located approximately 20 m from the borehole at -82.47047° -152.29072°. Vertical velocity was estimated using the gradient resulting from linear regression of the vertical position of the entire 2021 time series (Figure 1). Surface lowering at 1.52 $\pm$ 0.01 m a^{-1} was observed throughout 2021.

However, co-located repeat thickness observations show ongoing ice thinning is not driving this surface elevation change. Thickness change was estimated using repeat phase-sensitive Radio-Echo Sounding (pRES; Figure 2). Thickness and melt rate estimates (Supplementary Table 7) followed [7]. Note that negative basal mass balance reported in Supplementary Table 7 imply thickening by accretion but the magnitude of the accretion is more uncertain than indicated by the formal uncertainty [8]. We instead rely on the sign of the melt rate and thickness change, which are both the opposite of what would be expected if the surface lowering was associated with ongoing melt. This leads to the conclusion that the observed surface lowering is a result of the ice's ongoing viscous response to a previous melt event.

The channel surface shape downstream of our borehole consists of a series of basins underlain by wider channel cross sections [9], which can also be explained by discrete discharge events that resulted in additional melt when the channel head was at these locations. Episodic discharge events can also explain the morphology of the channel

cross section (Article Figures 1, 2A). Their enhanced discharge would ascend higher within the channel, entrain more seawater and be more turbulent, leading to greater melt resulting in the enlarged oblate upper cross section [10].

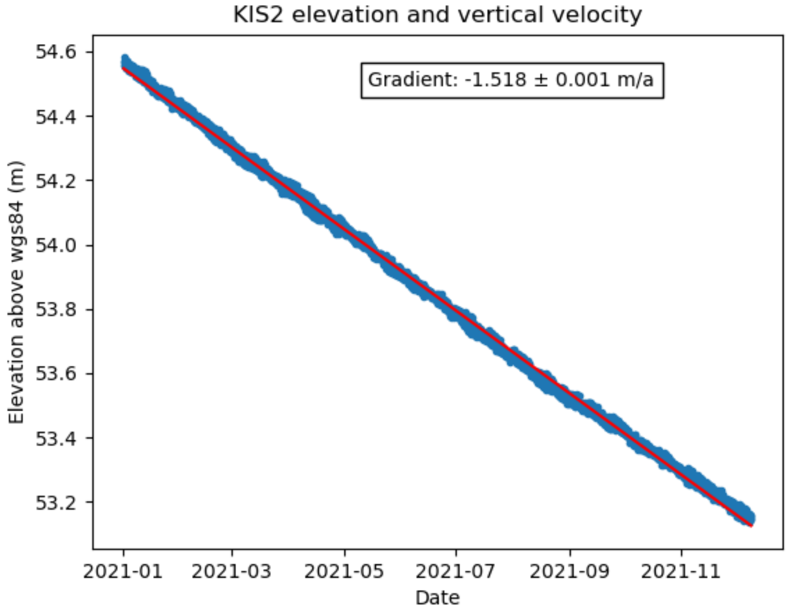

Supplementary Figure 1 GNSS observed height above WGS84 ellipsoid (blue) with best fitting linear regression overlain in red.

Observation 1	Observation 2	dt (days)	Thickness change (m)	Basal mass balance (m a^{-1})
2021-01-01	2021-02-01	31	0.17	-1.79 ± 0.21
2021-02-01	2021-03-01	28	0.16	-1.99 ± 0.10
2021-01-01	2021-03-01	59	0.32	-1.79 ± 0.21
2021-01-01	2021-04-01	90	0.47	-1.80 ± 0.24
2021-01-01	2021-12-31	364	2.35	-2.62 ± 0.39

Supplementary Table 7 Repeat phase sensitive radio echo sounding (pRES) derived thickness change and melt rate estimates.

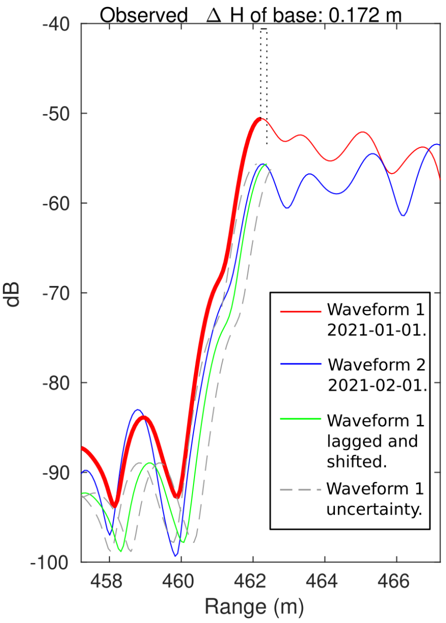

Supplementary Figure 2 Example of repeat ApRES range estimates from 2021-01-01 (red line) and 2021-02-01 (blue line). Green line denotes y-axis shifted and lagged version of first observation used to estimate the range difference. Grey dashed lines indicate half-wavelength uncertainty of this solution. Thickness change is estimated to be 0.172 m and internal strain is estimated as 0.019 m following [7]. Range distance is estimated using an ice velocity of 169 m μs^{-1}.

Modelled subglacial flux

Modelled subglacial flux was estimated using Monte Carlo simulations of the D8 routing method described in [11] and modelled basal melt production from [12]. Total subglacial flux was summed along an 18 km portion of the grounding zone encompassing the channel. Flux gates of 22 km and 31 km length were also tested resulting in no significant variance. Less than 1% of simulations resulted in subglacial flux of less than the observed 0.9 m^3s^{-1}. The distribution of flux estimates for the 18 km long flux gate is shown in Extended Data Figure E5. The bimodal distribution primarily results from simulated catchments favoring either the lower KIS (Extended Data Figure E5 Gaussian 1, mean flux of 6.3 m^3s^{-1}), or including the upper KIS (Extended Data Figure E5 Gaussian 2, mean flux 31.7 $m^3s^{-1}m^3s^{-1}$).

Downstream of the grounding zone channel a distributed region of ice shelf surface features and ice shelf melt is observed [Extended Data Figures E8, E9; 13]. Onshore, a complementary region of surface elevation change is observed using elevation data generated using swath processing of CryoSat-2 data [14] (Extended Data Figure 9). To estimate surface elevation change on Kamb Ice Stream, CryoSat-2 swath elevation data from July 2010 to December 2019 were spatially binned into 500 by 500 m tiles. An optimal dipping planar surface that allowed for a secular change in elevation with time was fitted to the elevation data in each bin by minimising the difference between the planar model and the observed elevations [15]. The resulting rate of surface elevation change is plotted upstream of the grounding zone in Extended Data Figure E9 and displays a surface down-wasting of -0.54 m/a at the borehole site.

References

[1] Farmer, G. L., Licht, K., Swope, R. J. & Andrews, J. Isotopic constraints on the provenance of fine-grained sediment in LGM tills from the Ross Embayment, Antarctica. *Earth and Planetary Science Letters* **249**, 90–107 (2006).

[2] Simkins, L. M. *et al.* Anatomy of a meltwater drainage system beneath the ancestral East Antarctic ice sheet. *Nature Geoscience* **10**, 691–697 (2017).

[3] Tulaczyk, S., Kamb, B., Scherer, R. P. & Engelhardt, H. F. Sedimentary processes at the base of a West Antarctic ice stream; constraints from textural and compositional properties of subglacial debris. *Journal of Sedimentary Research* **68**, 487–496 (1998).

[4] Kamb, B. in *Basal zone of the West Antarctic ice streams and its role in the lubrication of their rapid motion* (eds Alley, R. B. & Bindschadler, R. A.) *The West Antarctic Ice Sheet: Behavior and Environment*, Vol. 77 of *Antarctic Research Series* 157–200 (AGU, 2001).

[5] Lepp, A. P. *et al.* Sedimentary Signatures of Persistent Subglacial Meltwater Drainage From Thwaites Glacier, Antarctica. *Frontiers in Earth Science* **10** (2022).

[6] Balfoort, L. *Sedimentology and biomarker geochemistry of a Kamb Ice Stream subglacial channel, West Antarctica.* Master's thesis, Te Herenga Waka—Victoria University of Wellington (2023).

[7] Stewart, C. L., Christoffersen, P., Nicholls, K. W., Williams, M. J. M. & Dowdeswell, J. A. Basal melting of Ross Ice Shelf from solar heat absorption in an ice-front polynya. *Nature Geoscience* **12**, 435–440 (2019).

[8] Vaňková, I., Nicholls, K. W., Corr, H. F. J., Makinson, K. & Brennan, P. V. Observations of Tidal Melt and Vertical Strain at the Filchner-Ronne Ice Shelf, Antarctica. *Journal of Geophysical Research: Earth Surface* **125**, e2019JF005280 (2020).

[9] Whiteford, A., Horgan, H. J., Leong, W. J. & Forbes, M. Melting and Refreezing in an Ice Shelf Basal Channel at the Grounding Line of the Kamb Ice Stream, West Antarctica. *Journal of Geophysical Research: Earth Surface* **127**, e2021JF006532 (2022).

[10] Jenkins, A. Convection-Driven Melting near the Grounding Lines of Ice Shelves and Tidewater Glaciers. *Journal of Physical Oceanography* **41**, 2279–2294 (2011).

[11] Malczyk, G., Gourmelen, N., Werder, M., Wearing, M. & Goldberg, D. Constraints on subglacial melt fluxes from observations of active subglacial lake recharge. *Journal of Glaciology* 1–15 (2023).

[12] Van Liefferinge, B. & Pattyn, F. Using ice-flow models to evaluate potential sites of million year-old ice in Antarctica. *Climate of the Past* **9**, 2335–2345 (2013).

[13] Adusumilli, S., Fricker, H. A., Medley, B., Padman, L. & Siegfried, M. R. Interannual variations in meltwater input to the Southern Ocean from Antarctic ice shelves. *Nature Geoscience* **13**, 616–620 (2020).

[14] Gourmelen, N. *et al.* CryoSat-2 swath interferometric altimetry for mapping ice elevation and elevation change. *Advances in Space Research* **62**, 1226–1242 (2018).

[15] Smith, B. E., Fricker, H. A., Joughin, I. R. & Tulaczyk, S. An Inventory of active subglacial lakes in Antarctica detected by ICESat (2003–2008). *Journal of Glaciology* **55**, 573–593 (2009).
