# Supplementary figures and images for: A West Antarctic grounding-zone environment shaped by episodic water flow
Source: Nat Geosci. 2025 May 12;18(5):389–95. doi: 10.1038/s41561-025-01687-3 (PMC12075004; doi:10.1038/s41561-025-01687-3)

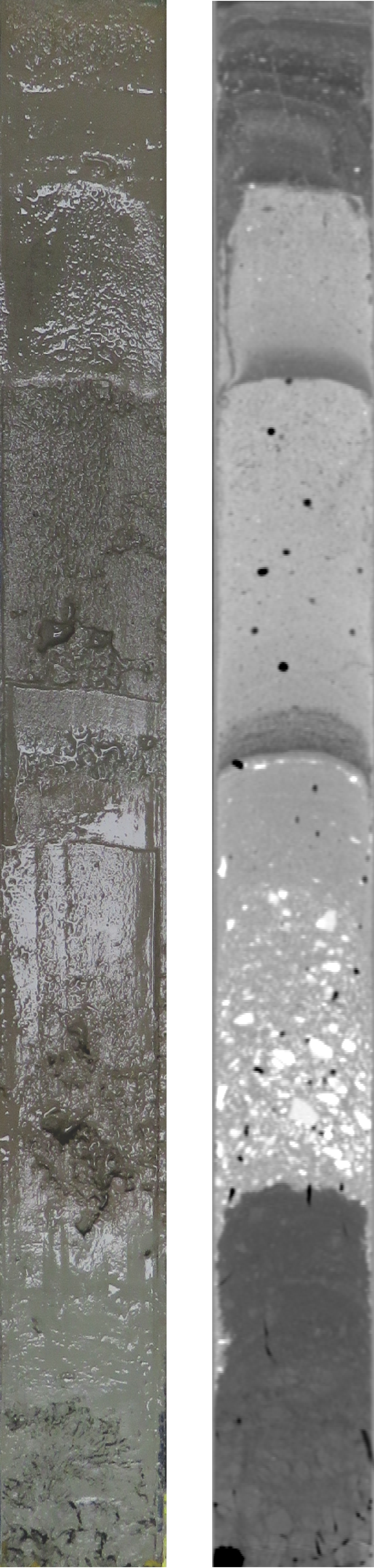

Supplement: Supplementary file 5 — Image of sediment core and CT scan (KIS2Sed_nolabels_v3.tif), provenance data (kis2provenance.csv), grain-size data (kis2Grainsize.csv) and density data (kis2CTdensity.csv). [file 41561_2025_1687_MOESM5_ESM.zip › KIS2Sed_nolabels_v3.tif]

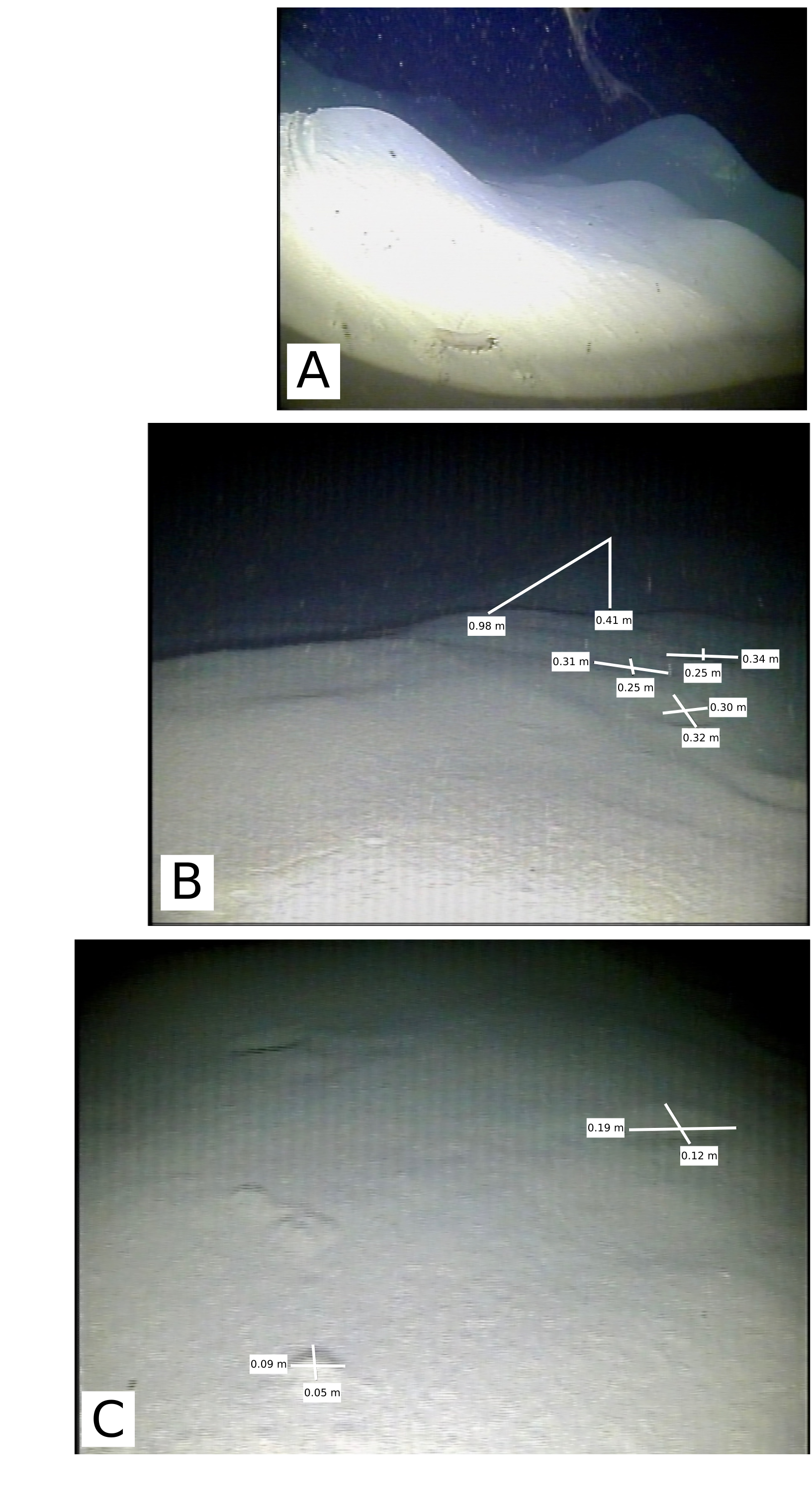

Supplement: Supplementary file 10 — Channel-floor imagery of dropstones. [file 41561_2025_1687_MOESM10_ESM.zip › dropstone_summary.tif]
